# Anti-Melanogenic Property of Geoditin A in Murine B16 Melanoma Cells

**DOI:** 10.3390/md10020465

**Published:** 2012-02-16

**Authors:** Florence W. K. Cheung, Jia Guo, Yick-Hin Ling, Chun-Tao Che, Wing-Keung Liu

**Affiliations:** 1 School of Biomedical Sciences, Faculty of Medicine, The Chinese University of Hong Kong, Shatin, New Territories, Hong Kong, China; Email: florence.cheung@cuhk.edu.hk (F.W.K.C.); guojia@cuhk.edu.hk (J.G.); lf2lyhs@hotmail.com (Y.-H.L.); 2 Department of Medical Chemistry & Pharmacognosy, College of Pharmacy, University of Illinois at Chicago, 833 S. Wood Street, Chicago, IL 60612, USA; Email: chect@uic.edu

**Keywords:** geoditin A, B16 melanoma cells, melanogenesis, tyrosinase

## Abstract

Geoditin A, an isomalabaricane triterpene isolated from marine sponge *Geodia japonica*, has been demonstrated to induce apoptosis in leukemia HL60 cells and human colon HT29 cancer cells through an oxidative stress, a process also interfering with normal melanogenesis in pigment cells. Treatment of murine melanoma B16 cells with geoditin A decreased expression of melanogenic proteins and cell melanogenesis which was aggravated with adenylate cyclase inhibitor SQ22536, indicating melanogenic inhibition was mediated through a cAMP-dependent signaling pathway. Immunofluorescence microscopy and glycosylation studies revealed abnormal glycosylation patterns of melanogenic proteins (tyrosinase and tyrosinase-related protein 1), and a co-localization of tyrosinase with calnexin (CNX) and lysosome-associated membrane protein 1 (LAMP-1), implicating a post-translational modification in the ER and a degradation of tyrosinase in the lysosome. Taken together, potent anti-melanogenic property and the relatively low cytotoxicity of geoditin A have demonstrated its therapeutic potential as a skin lightening agent.

## 1. Introduction

Melanocytes are the major cell population in the skin epidermis which is responsible for melanin production and pigmentation of skin and hair. Melanin is synthesized in melanocytes upon ultraviolet irradiation and then transported to surrounding epidermal keratinocytes for absorption of energy and protection from sunburn. However, abnormal melanogenesis causes pigmentary disorders, such as hyperpigmentation (melasma and lentigines) or hypopigmentation (vitiligo and albinism), which are of clinical and cosmetic concerns. Melanogenic agents are commonly employed to modulate melanogenesis. Many widely used lightening compounds such as kojic acid or arbutin are isolated from natural sources or botanical extracts [1]; few were of marine origin [2], despite its tremendous biological and structural diversity [3]. 

Melanogenesis is the process of pigment formation initiated by the rate-limiting enzyme tyrosinase that converts L-DOPA to dopaquinone before further oxidation to melanin. Activation of tyrosinase is associated with phosphorylation by protein kinase C-beta (PKC-β) and formation of a complex between phosphorylated tyrosinase and tyrosinase-related protein 1 (TYRP1) [4]. In melanocytes, converging external stimuli stimulates adenylyl cyclase to raise cAMP that subsequently activates microphthalmia-associated transcription factor (MITF), a transcription factor for all melanogenic proteins, including tyrosinase (TYR), TYRP1, tyrosinase-related protein 2 (TYRP2), and PKC-β. MITF is thus a key factor linking both PKC-β- and cAMP-dependent pathways in the regulation of melanogenesis [5]. Reduction of MITF binding to the promoters of these melanogenic proteins results in a decrease of cell pigmentation. Furthermore, MITF is also the master regulator for DNA replication, proliferation and genomic stability of melanoma cells and its reduction leads to DNA damage and defective cell replication [6]. The precise function(s) of TYRP2 in melanogenesis, a DOPA-chrome tautomerase, remains largely unknown although it decreases cell sensitivity to oxidative stress and also modulates melanoma cell sensitivity to chemotherapy [4,7]. 

Reactive oxygen species (ROS) are not only by-products of normal cell metabolism but also act as signaling molecules in cell functions. An excessive ROS production causes damage of DNA, proteins and lipids, ultimately resulting in apoptosis and cell death. In melanocytes, intracellular ROS at sublethal concentrations induce melanogenesis [8] through activation of apurinic/apyrimidinic endonuclease (APE) for oxidative DNA damage repair [9,10] and promotion of cell survival via MITF pathway [9]. Interestingly, kojic acid, a widely used skin whitening agent, possesses both anti-tyrosinase and ROS scavenging activities [11]. 

Geoditin A, an isomalabaricane triterpenoid isolated from many genera of marine sponges, has received special pharmaceutical attention because it inhibited cyclin-dependent kinase activity and subsequently suppressed tumor cell proliferation [12,13]. Our previous studies have also demonstrated geoditin A being a potent inducer of oxidative stress and apoptosis in HT29 cells [14] and HL60 cells [15]. It is the objective in this study to investigate if this oxidative stress might interfere with tyrosinase activity and melanogenesis in murine B16 melanoma cells.

## 2. Results and Discussion

**Figure 1:**
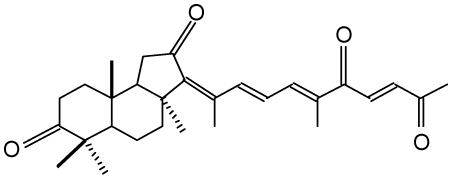
Chemical structure of geoditin A.

Geoditin A (Figure 1) is an isomalabaricane triterpene isolated from a South China Sea sponge, *Geodia japonica* [16]. This rare class of triterpenoids possesses potent tumor inhibitory activities [17,18], and thus received special attention for its potential for chemotherapeutic development [19]. 

**Figure 2:**
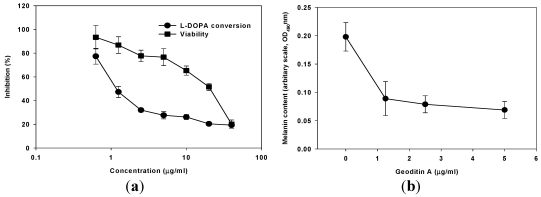
(**a**) Viability and melanogenic activity of geoditin A on murine melanoma B16 cells. Cells were incubated in 96-well plates with serial concentrations of geoditin A for 48 h, and their viability was assessed by sulforhodamine B (SRB) colorimetric assay. Another set of cells were lysed with 20 mM Tris base (pH 7) and reacted with L-DOPA for cellular tyrosinase activity (*i.e.*, conversion of L-DOPA into dopaquinone) assay. Results were presented as mean and standard deviation of triplicates. (**b**) Melanin in cell lysates was pelleted and extracted with 1 M NaOH before the optical density absorbance of melanin solutions was determined at 490 nm. The melanin contents normalized to cellular protein content decrease in a dose-dependent manner. Results were presented as mean and standard deviation of triplicates.

**Figure 3:**
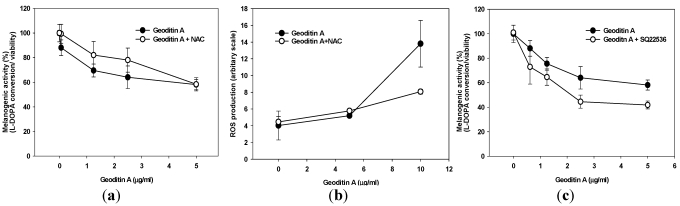
(**a**) Melanogenic activity (=ratio of L-DOPA conversion to viable cells) by geoditin A on B16 cells. Cells incubated in 96-well plates with geoditin A, ranging from 0.625 to 5 μg/mL, for 48 h was only slightly attenuated by pre-treatment with 10 µM *N*-acetylcysteine (oxidant scavenger). (**b**) Flow cytometric analysis revealed a low level of ROS production in cells treated with geoditin A at doses ≤5 µg/mL until an about 3.5-fold increase was measured at dose of 10 µg/mL whose production was greatly attenuated in the presence of 10 µM of NAC. Results were presented as mean and standard deviation of triplicates. (**c**) Inhibition of intracellular cyclic adenosine monophosphate (cAMP) by pre-incubation for 30 min with specific inhibitor SQ22536 (100 µM) further reduced melanogenic activity, indicating cAMP pathway is involved in geoditin-A mediated melanogenesis.

The values of IC_50_ for cancer cell lines ranging from 0.1–20 µg/mL which were 2–3 folds higher than that for human fibroblasts (IC_50_ = 60 μg/mL) [14]. Our previous study has also demonstrated a potent cytotoxicity of geoditin A against human leukemia HL60 cells (IC_50_ = 3 µg/mL) [15], but mild to human colon carcinoma HT29 cells (IC_50_ = 20 μg/mL) [14]. The IC_50_ for murine melanoma B16 cells in this study also falls in this range, with the value of ≈10 μg/mL (Figure 2a). In addition to its cytotoxicity, geoditin A also interfered with L-DOPA conversion activity, a parameter indicative of melanogenesis, which decreased dramatically from 80% at 0.625 µg/mL to 25% at 5 µg/mL, with an EC_50_ value of about 1 μg/mL. EC_50_ value is defined as the effective concentration of geoditin A required to inhibit half maximal inhibition of L-DOPA conversion, and the ratio of L-DOPA conversion to cell viability is defined as melanogenic activity (Figure 3a). Since an effective activity was achieved between 1.25 µg/mL to 5 µg/mL when most of the tested cells (≥80%) remained viable, the following study was therefore focused on this range.

Instead of using MTT cytotoxicity assay for HL60 cells and HT29 cells in previous studies, sulforhodamine B (SRB) colorimetric viability assay [20] was used in this study because the absorbance wavelength of the melanin might interfere with that of methyl tetrazolium for MTT assay. In consistent with the prominent decrease of L-DOPA conversion activity in B16 cells after geoditin A treatment, levels of intracellular melanin content, the end product of this melanogenesis, also reduced in a dose-dependent manner (Figure 2b).

Since geoditin A is an oxidative stressor and ROS is closely associated with melanogenesis, it is tempting to speculate that oxidative stress may interfere with pigmentation in B16 cells. This inhibitory effect can partially be attenuated by pre-treatment with 10 µM *N*-acetylcysteine (NAC) (Figure 3a), an oxidative scavenger also effectively reducing apoptosis in HT29 cells [14]. Interestingly, no noticeable ROS production was measured until B16 cells were exposed to higher doses of geoditin A (≥10 µg/mL) thereby ROS production increased by about 3.5 folds (Figure 3b), which suggested that ROS might not contribute to anti-melanogenic activity of geoditin A at doses ≤5 µg/mL. On the other hands, anti-melanogenesis was aggravated by SQ22536, an adenylate cyclase inhibitor (Figure 3c); implicating that geoditin A-mediated melanogenesis is regulated through modulation of cAMP pathway. When excessive ROS production overwhelms cell tolerance at doses of geoditin A ≥10 μg/mL, cells committed to cell death (Figure 2a), probably from apoptosis induced by oxidative stress as happened to HT29 cells and HL60 cells [14,15].

**Figure 4:**
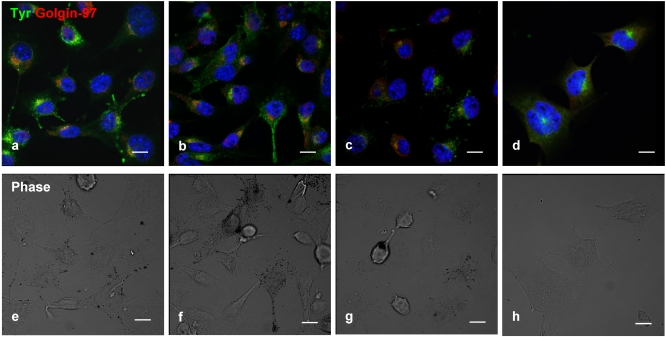
Dual immunofluorescence localization of tyrosinase with the Golgi marker in B16 cells. Cells were treated with serial concentrations of geoditin A for 48 h, fixed in 3% paraformaldehyde and incubated with an antibody against golgin-97 (**a**–**d**), the marker protein for the Golgi apparatus. Immunofluorescence was visualized by secondary antibody IgG conjugated to Alexa-488 (green) and Alexa-594 (red). Nuclei were counterstained with DAPI (blue). Typical Golgi stack (golgin-97, red) is shown at the juxtanuclear region while tyrosinase (green) in the cytoplasm of the untreated B16 cells (**a**) which was decreased in a dose-dependent manner (**b**, **c**, and **d** for 0.6, 1.25 and 5 μg/mL, respectively). Melanin granules also decreased in a dose-dependent manner after geoditin A treatment (**e**–**h**) under the phase contrast microscope (scale bar = 100 μm). No apoptotic bodies were found in DAPI-stained cells of all treatment groups (0.6–5 μg/mL)(**b**–**d**).

Double-label immunofluorescence microscopy has demonstrated a dose-dependent decrease of tyrosinase (Figure 4a–d) and cytosolic melanin (Figure 4e–h) in B16 cells treated with geoditin A, particularly at dose of 5 μg/mL (Figure 4d,h). No co-localization of tyrosinase with golgin-97, marker protein for the Golgi, indicate retention of tyrosinase in this organelle is unlikely (Figure 4b–d).

**Figure 5:**
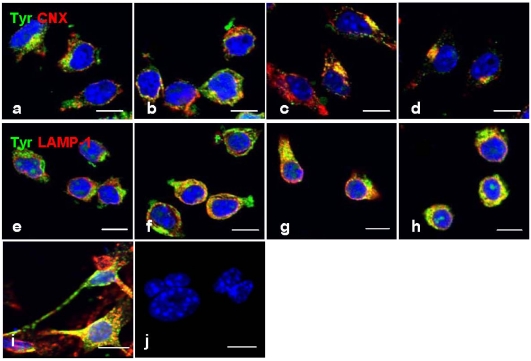
Co-localization immunofluorescence of tyrosinase in ER and lysosomes. Cells were treated with geoditin A [untreated (**a**), 1.25 μg/mL (**b**), 2.5 μg/mL (**c**), 5 μg/mL (**d**) for 48 h], fixed with 3% paraformaldehyde and immunostained with antibodies against tyrosinase (green), CNX protein (red) in the ER (**a**–**d**) and LAMP-1 (red) in the lysosome (**e**–**h**). No retention of tyrosinase in the ER and lysosomes as depicted by two separated immunostains in cells without geoditin A treatment (**a**,**e**), but strong co-localization (yellow) was observed in cells with geoditin A treatment (**b**–**d** and **f**–**h** for CNX and LAMP-1 with tyrosinase in the ER and lysosomes, respectively). A co-localized tyrosinase and LAMP-1 was also shown in melan-a cells treated with progesterone for 4 days as a positive control (**i**).Apoptotic nuclei were observed in DAPI-stained cells treated with higher dose of geoditin A (10 μg/mL) for 48 h (scale bar = 100 μm).

However, a strong immunofluorescence tyrosinase was co-localized with CNX (Figure 5b–d) and LAMP-1 (Figure 5f–h), not only indicating its retention in the ER and lysosomes, but also a possibility of improper trafficking of this enzyme to the lysosome for degradation. This phenomenon was also found in mouse melanocyte melan-a cells treated with progesterone (Figure 5i) [21], or B16 cells treated with inulavosin, a flavanol isolated from Inula nervosa [22].

The initial catalytic reaction of melanogenesis (a physiological process of melanin biosynthesis) is regulated by the rate-limiting enzyme, tyrosinase, and its related proteins, TYRP1 and TYRP2 [23]. Tyrosinase is activated when it is glycosylated, phosophorylated and forming a complex with TYRP1 in organelles along the melanogenic pathway, namely endoplasmic reticulum, the Golgi and the melanosome [4,24]. However, tyrosinase has common targeting motifs for melanosomes and lysosomes, making it possible to be mistargeted to lysosomes for degradation [25]. Whether this is a result of trafficking defect or improper maturation of tyrosinase in geoditin A-treated cells remains unclear in this study, and further study is required to elucidate its underlying mechanism.

Members of melanogenic genes, including tyrosinase, TYRP1, and PKC-β, are regulated at their transcriptional levels by microphthalmia-associated transcription factor (MITF) [5]. In addition to pigmentation, MITF also promotes pigment cell growth and differentiation [26]. MITF expression is activated by agents that elevate the intracellular level of cAMP [5] or apurinic/apyrimidinic endonuclease (APE-1/Ref-1) in response to ROS [10], and thus strong oxidative stress and cAMP inhibitors impinging on MITF become targets for depigmentation. Resveratrol is a good example for post-translational regulation through cAMP pathway [25].

**Figure 6:**
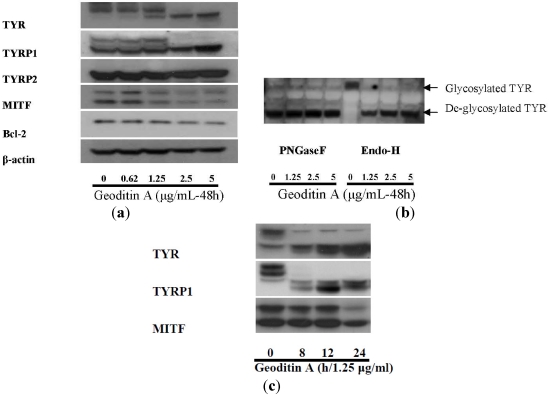
(**a**) Expression of melanogenic genes in geoditin A-treated B16 cells. Protein lysates of B16 cells treated with geoditin A, ranging from 0 to 5 μg/mL, for 48 h were subject to western blotting for examination of levels of melanogenic proteins (TYR, TYRP1, TYRP2 and MITF). Images represent results from at least three separated experiments. Equal amount of proteins analyzed were normalized with β-actin. (**b**) Glycosylation study of tyrosinase with EndoH and PGNase F digestion shows matured tyrosinase was present in untreated B16 cells while aberrant maturation of tyrosinase increased in a dose-dependent manner after treatment with geoditin A (1.25, 2.5 and 5 μg/mL) for 48 h. (**c**) Western blotting for kinetic analysis of melanogenic protein expression in B16 cells treated with 1.25 μg/mL geoditin A for 8–24 h. Two bands for tyrosinase and three bands for TYRP1 were present in B16 cells, proteins of larger size (~80 kD) represents mature tyrosinase/TYRP1 while bands of smaller size represent immature glycoforms of these two proteins. Immature TYR and TYRP1 were prominent after treatment of 1.25 g/mL geoditin A for 8 h, while decrease of MITF expression remained at untreated levels until 24 h, which is far behind the change of both TYR and TYRP1.

The decrease of immunofluorescence tyrosinase in B16 cells (Figure 4 and Figure 5) after geoditin A treatment is further confirmed by immunoblotting for melanogenic proteins (Figure 6). One major band for tyrosinase (80 kD) was revealed in untreated B16 cells while a second band for protein of smaller size (about 70 kD) increased in a dose-dependent manner in geoditin A-treated cells (Figure 6a). These bands represent different degrees of maturation of melanogenic proteins (tyrosinase and tyrosinase-related proteins) in the ER [27]. Nascent tyrosinase is glycosylated and properly folded in the ER and completely matured in the Golgi before it is secreted to melanosomes for melanogenesis. Immature tyrosinase retains in the ER and bound to chaperones, such as calnexin (CNX) and calreticulin. Thus, our immunofluorescence analysis shows a reduced amount of tyrosinase in the Golgi and cytoplasm but it bound significantly with CNX, implicating retention of immature tyrosinase in the ER (Figure 5b–d). Similar immunoblotting pattern was also found for TYRP1 protein. This decrease was not attributed to cell death because there were still about 80% viable cells at 5 μg/mL when the L-DOPA conversion activity had already reduced to only 30% of the untreated control (Figure 2a).

The *N*-glycan processing of tyrosinase in the ER is noteworthy because the immature form increased with the expense of its mature forms but the total amount of these proteins did not change significantly (Figure 6). The oligosaccharide added to tyrosinase in the ER can be cleaved by endoglycosidase H (EndoH), while those added in both ER and the Golgi can be cleaved by peptide *N*-glycosidase F (PNGase F) [25]. The analysis of tyrosinase pattern by these two endoglycosidases demonstrates a strong band with higher molecular weight for mature tyrosinase but an absence of immature tyrosinase only in untreated cells. On the other hand, immature and EndoH-sensitive glycoforms of tyrosinase increased in B16 cells after geoditin A treatment (Figure 6b), implicating aberrant glycosylation was induced by geoditin A that affected melanogenesis of B16 cells.

Since MITF is a master transcription factor for members of melanogenic proteins, its down-regulation should have a direct inhibition on the expression of TYR and TYRP1. However, a kinetic analysis of expression of these three proteins in cells treated with 1.25 μg/mL geoditin A (≈EC_50_) for 8–24 h has revealed a significant reduction of expression of TYR and TYRP1 at 8 h which preceded the decrease of MITF at 12–24 h, indicating the decrease of TYR and TYRP1 is independent of MITF, and two separated pathways might be involved in their decrease (Figure 6c).

Apart from melanogenic transcription of tyrosinase, MITF also regulates melanocytic proliferation and survival by up-regulating expression of anti-apoptotic bcl-2 gene as well as a number of survival genes including Pax3, c-kit, Sox9, Sox10 and LEF1 [10]. A depletion of MITF causes defective replication and cell senescence and death [6]. B16 cells died with increasing doses of geoditin A when the production of oxidants were overwhelmed (Figure 3b) and the level of anti-apoptotic Bcl-2 protein decreased in a dose-dependent manner (Figure 6a). Cells probably died by an apoptotic mechanism (Figure 5j). 

Of the four marine sponge isomalabaricane triterpenes, namely stellettins A and B, and geoditins A and B, tested in HL60 and HT29 cells, the carbonyl group at C-3 position of geoditin A was responsible for its strong apoptotic induction activity [18]. However doses of geoditin A inhibiting tyrosinase activity (IC_50_ ≈ 1 μg/mL) far precede those for cell death/apoptosis (≥10 μg/mL), implicating a machinery other than apoptosis induction by the carbonyl group may contribute to the down-regulation of MITF protein and tyrosinase activity. Experiment is being conducted to elucidate the molecular mechanism underlying the anti-tyrosinase activity elicited by geoditin A.

## 3. Experimental Section

### 3.1. Test Compounds

Geoditin A (MW = 450), an isomalabaricane triterpene isolated from a marine sponge, *Geodia japonica* [10,16], was dissolved in DMSO to make a stock solution at a concentration of 40 mg/mL which was then diluted to appropriate concentrations with culture medium before each experiment. The final concentration of DMSO did not exceed 0.5% in any experiment.

### 3.2. Cell Cultures

Murine B16F10 melanoma cells (CRL6475, ATCC) were cultured in DMEM medium supplemented with 10% fetal bovine serum (FBS), 100 µg/mL streptomycin and 100 IU/mL penicillin at 37 °C in a humidified atmosphere of 5% CO_2_.

### 3.3. Cell Cytotoxicity Assay

Sulforhodamine B (SRB) colorimetric cytotoxicity assay was used in this study [20]. Briefly, B16 cells (5000 cells/0.1 mL/well) were treated with a serial dilution of geoditin A (0.6, 1.25, 2.5 and 5 μg/mL) in 96-well plates (Costar, USA) for 48 h, the reaction was stopped, the proteins were fixed by 50% trichloroacetic acid for 30 min at 4 °C, and stained with 0.4% SRB in 1% acetic acid for 30 min at room temperature. The unbound dye was removed and the cells were rinsed with 1% acetic acid for five times, air-dried and the bound dye was dissolved in 100 µL of Tris base (10 mM, pH 10.5). The absorbance was measured with a microtiter plate reader (Molecular Devices, Model Emax) at 570 nm. Data represent the mean values and standard deviations of triplicate assays in at least three separate experiments. Cells pre-treated with oxidative scavenger, *N*-acetylcysteine (NAC) and adenylyl cyclase inhibitor SQ22536 [9-(tetrahydro-2-furanyl)-9H-purin-6-amine] were used to study the roles of oxidative stress and activation of cAMP in geoditin A-mediated anti-melanogenesis [27]. 

### 3.4. Production of Reactive Oxygen Species

Sensitive probe, 2′,7′-dichlorodihydrofluorescein diacetate (H_2_DCF-DA, D399 Molecular Probes, Inc., Oregon, USA) was used in this study to analyze ROS induced by geoditin A treatment. Briefly, B16 cells were plated at 3 × 10^5^/750 µL supplemented RPMI medium in each well of a 6-well culture plate overnight, and the cultures were then incubated in PBS containing 10 µM H_2_DCF-DA for 15 min at 37 °C before being stimulated with various doses of geoditin A for 4 h. The intensity of the fluorescence for reactive species was measured using a flow cytometer at excitation/emission at 488/535 nm. Cells pre-incubated or co-incubated with NAC (10 μM) and SQ22536 (100 μM) before geoditin A treatment were used to elucidate the sources of ROS and cAMP production [27].

### 3.5. L-DOPA Conversion Assay

B16 cells were treated with geoditin A for 48 h before they were rinsed briefly with warm phosphate buffer saline (PBS). Cells were then lysed in 100 μL of 20 mM Tris containing 0.1% triton x-100, pH7.0, and reacted with equal volume of 1 mg/mL L-DOPA at 37 °C for 4 h. The absorbance at 490 nm of the reacted mixture for the tyrosinase activity was measured with a microtiter plate reader (Molecular Devices, Model Emax) and expressed as percent of untreated controls. 

Part of the cell lysates was used for melanin content measurement [28]. The lysates were centrifuged; protein concentrations of the supernatants were determined using BCA protein assay kit (Thermo Scientific Pierce). The pellets were solubilized in 1 M NaOH at 50 °C for 1 h, and the optical density absorbance for melanin content was determined at 490 nm with a microtiter plate reader. The relative melanin content was normalized to equal amount (μg) of protein. 

### 3.6. Fluorescence Staining for Morphological Observation

B16 cells were seeded on sterile coverslips and treated with a serial dilution of geoditin A for 48 h, washed briefly with PBS before they were fixed with paraformaldehyde, stained with antibodies against (1) tyrosinase, (2) golgin-97, a marker for the Golgi complex, and (3) calnexin, a lectin chaperone bound to the nascent tyrosinase after the addition of two glycans in the ER, followed by secondary antibody IgG conjugated to AlexaFluor-488 or AlexaFluor-594 (Figure 4 and Figure 5) in PBS buffer. The chromatin was counter-stained with DAPI before the slides were mounted with anti-fade for confocal microscopy (Axioskop, Zeiss, Japan) with a 450–490 nm excitation block filter and a 520 nm barrier filter [15]. 

### 3.7. Immunoblotting Analysis

B16 cells after 48 h drug exposure were washed with PBS twice, and the total protein lysates were obtained in lysis buffer (50 mM Tris-Cl, 150 mM NaCl, 0.2% Triton X-100, 10 µg/mL aprotinin and 0.5 mM PMSF), and centrifuged at 10,000 rpm at 4 °C for 10 min. Lysates were normalized for protein content using the protein assay reagent (500-0006, Bio-Rad Laboratories, USA). Equal amounts of denatured proteins were loaded and separated on a 10% SDS polyacrylamide gel, and were then transferred onto a polyvinylene difluoride (PVDF) membrane. After blocking with 2% gelatin, the membrane was stained with specific primary antibodies against melanogenic proteins: tyrosinase (13-6800 Zymed Laboratories, South San Francisco, CA, USA), tyrosinase-related protein-1 (TYRP1, a kind gift of Dr. Vincent Hearing, National Institutes of Health, Bethesda, MD, USA), TYRP2 (sc-25544 Santa Cruz Biotechnology, Santa Cruz, CA, USA), microphthalmia-associated transcription factor (MITF, MS-771 Thermo Scientific, USA), bcl-2 (sc492, Santa Cruz Biotechnol.) and β-actin (sc-81178 Santa Cruz Biotechnol.), respectively, followed by secondary antibody IgG conjugated to horseradish peroxidase in TBS-T buffer. The signals were detected using the ECL™ Plus Western Blotting Analysis System (GE healthcare Bio-Sciences, Piscataway, NJ, USA), followed by short exposures to Lumi-film Chemiluminescence Detection Film (Roche Diagnostics Corporation, Indianapolis, IN, USA). Band intensities were quantified by the software PD Quest (BioRad Laboratories, Hercules, CA, USA) and normalized by β-actin [15]. 

### 3.8. Glycosylation Analysis

Proteins were extracted as described in 3.7. and 10 μg of protein lysates were subjected to enzyme digestion by either 100 units of endoglycosidase H (EndoH, P0702S, New England Biolabs, Beverly, MA, USA) or 100 units of peptide *N*-glycosidase F (PNGase F, P0704S, New England Biolabs) at 37 °C for 1 h according to the conditions specified in the manufacturer’s instruction (New England Biolabs). After incubation, the digested protein lysates were separated on a 8% gel and subjected to Western blotting using anti-tyrosinase antibody.

## 4. Conclusions

Geoditin A at sublethal doses (≤5 μg/mL) decreased melanogenesis and glycosylation of tyrosinase (TYR) in murine B16 melanoma cells in a dose-dependent, but ROS- and MITF-independent manner. There is potential in application and development of this marine compound as a skin lightening agent.

## References

[1] Zhu W., Gao J. (2008). The use of botanical extracts as topical skin-lightening agents for the improvement of skin pigmentation disorders. J. Invest. Dermatol. Symp. Proc..

[2] Heo S.J., Ko S.C., Cha S.H., Kang D.H., Park H.S., Choi Y.U., Kim D., Jung W.K., Jeon Y.J. (2009). Effect of phlorotannins isolated from *Ecklonia cava* on melanogenesis and their protective effect against photo-oxidative stress induced by UV-B radiation. Toxicol. Vitro.

[3] Molinski T.F., Dalisay D.S., Lievens S.L., Saludes J.P. (2009). Drug development from marine natural products. Nat. Rev. Drug Discov..

[4] Wu H., Park H.Y. (2003). Protein kinase C-beta-mediated complex formation between tyrosinase and TRYP1. Biochem. Biophys. Res. Commun..

[5] Park H.Y., Wu C., Yonemoto L., Murphy-Smith M., Wu H., Stachur C.M., Gilchrest B.A. (2006). MITF mediates cAMP-induced protein kinase C-beta expression in human melanocytes. Biochem. J..

[6] Strub T., Giuliano S., Ye T., Bonet C., Keime C., Kobi D., Le Gras S., Cormont M., Ballotti R., Bertolotto C. (2011). Essential role of microphthalmia transcription factor for DNA replication, mitosis and genomic stability in melanoma. Oncogene.

[7] Pak B.J., Lee J., Thai B.L., Fuchs S.Y., Shaked Y., Ronai Z., Kerbel R.S., Ben-David Y. (2004). Radiation resistance of human melanoma analysed by retroviral insertional mutagenesis reveals a possible role for dopachrome tautomerase. Oncogene.

[8] Zhao Y., Liu J., McMartin K.E. (2008). Inhibition of NADPH oxidase activity promotes differentiation of B16 melanoma cells. Oncol. Rep..

[9] Yang S., Misner B.J., Chiu R.J., Meyskens F.L. (2007). Redox effector factor-1 combined with reactive oxygen species, plays an important role in the transformation of JB6 cells. Carcinogenesis.

[10] Liu F., Fu Y., Meyskens F.L. (2009). MiTF regulates cellular response to reactive oxygen species through transcriptional regulation of APE-1/Ref-1. J. Invest. Dermatol..

[11] Niwa Y., Akamatsu H. (1991). Kojic acid scavenges free radicals while potentiating leukocyte functions including free radical generation. Inflammation.

[12] Singh R., Sharma M., Joshi P., Rawat D.S. (2008). Clinical status of anti-cancer agents derived from marine sources. Anticancer Agents Med. Chem..

[13] Tasdemir D., Mangalindan G.C., Concepción G.P., Verbitski S.M., Rabindran S., Miranda M., Greenstein M., Hooper J.N., Harper M.K., Ireland C.M. (2002). Bioactive isomalabaricane triterpenes from the marine sponge *Rhabdastrella globostellata*. J. Nat. Prod..

[14] Cheung F.W., Li C., Che C.T., Liu B.P., Wang L., Liu W.K. (2010). Geoditin A induces oxidative stress and apoptosis on human colon HT29 cells. Mar. Drugs..

[15] Liu W.K., Ho J.C., Che C.T. (2005). Apoptotic activity of isomalabaricane triterpenes on human promyelocytic leukemia HL60 cells. Cancer Lett..

[16] Zhang W.H., Che C.T. (2001). Isomalabaricane-type nortriterpenoids and other constituents of the marine sponge *Geodia japonica*. J. Nat. Prod..

[17] Clement J.A., Li M., Hecht S.M., Kingston D.G. (2006). Bioactive isomalabaricane triterpenoids from *Rhabdastrella globostellata* that stabilize the binding of DNA polymerase beta to DNA. J. Nat. Prod..

[18] Lin H.W., Wang Z.L., Wu J.H., Shi N., Zhang H.J., Chen W.S., Morris-Natschke S.L., Lin A.S. (2007). Stellettins L and M, cytotoxic isomalabaricane-type triterpenes, and sterols from the marine sponge *Stelletta tenuis*. J. Nat. Prod..

[19] Meragelman K.M., McKee T.C., Boyd M.R. (2001). New cytotoxic isomalabaricane triterpenes from the sponge *Jaspis* Species. J. Nat. Prod..

[20] Kim H.M., Han S.B., Kim M.S., Kang J.S., Oh G.T., Hong D.H. (1996). Efficient fixation procedure of human leukemia cells in sulforhodamine B cytotoxicity assay. J. Pharmacol. Toxicol. Methods.

[21] Hall A.M., Krishnamoorthy L., Orlow S.J. (2003). Accumulation of tyrosinase in the endolysosomal compartment is induced by U18666A. Pigment Cell Res..

[22] Fujita H., Motokawa T., Katagiri T., Yokota S., Yamamoto A., Himeno M., Tanaka Y. (2009). Inulavosin, a melanogenesis inhibitor, leads to mistargeting of tyrosinase to lysosomes and accelerates its degradation. J. Invest. Dermatol..

[23] Wang N., Hebert D.N. (2006). Tyrosinase maturation through the mammalian secretory pathway: Bringing color to life. Pigment Cell Res..

[24] Kobayashi T., Hearing V.J. (2007). Direct interaction of tyrosinase with Tyrp1 to form heterodimeric complexes *in vivo*. J. Cell Sci..

[25] Newton R.A., Cook A.L., Roberts D.W., Leonard J.H., Sturm R.A. (2007). Post-transcriptional regulation of melanin biosynthetic enzymes by cAMP and resveratrol in human melanocytes. J. Invest. Dermatol..

[26] Lekmine F., Chang C.K., Sethakorn N., Das Gupta T.K., Salti G.I. (2007). Role of microphthalmia transcription factor (MITF) in melanoma differentiation. Biochem. Biophys. Res. Commun..

[27] Negroiu G., Branza-Nichita N., Petrescu A.J., Dwek R.A., Petrescu S.M. (1999). Protein specific *N*-glycosylation of tyrosinase and tyrosinase-related protein-1 in B16 mouse melanoma cells. Biochem. J..

[28] Cheung F.W.K., Che C.T., Sakagami H., Kochi M., Liu W.K. (2010). Sodium 5,6-benzylidene-L-ascorbate induces oxidative stress, autophagy and growth arrest in human colon cancer HT-29 cells. J. Cell. Biochem..

